# A Partial C_4_ Photosynthetic Biochemical Pathway in Rice

**DOI:** 10.3389/fpls.2020.564463

**Published:** 2020-10-15

**Authors:** HsiangChun Lin, Stéphanie Arrivault, Robert A. Coe, Shanta Karki, Sarah Covshoff, Efren Bagunu, John E. Lunn, Mark Stitt, Robert T. Furbank, Julian M. Hibberd, William Paul Quick

**Affiliations:** ^1^C_4_ Rice Centre, International Rice Research Institute (IRRI), Los Baños, Philippines; ^2^Max Planck Institute of Molecular Plant Physiology (MPI-MP), Potsdam, Germany; ^3^National Centre for Fruit Development, Kirtipur, Nepal; ^4^Department of Plant Sciences, University of Cambridge, Cambridge, United Kingdom; ^5^ARC Centre of Excellence for Translational Photosynthesis, Research School of Biology, The Australian National University, Acton, ACT, Australia; ^6^Department of Animal and Plant Sciences, University of Sheffield, Sheffield, United Kingdom

**Keywords:** C_4_ rice, C_4_ photosynthesis, ^13^C labeling, NADP-malic enzyme, malate, *Oryza sativa* (rice), transgenic rice, metabolic engineering

## Abstract

Introduction of a C_4_ photosynthetic pathway into C_3_ rice (*Oryza sativa*) requires installation of a biochemical pump that concentrates CO_2_ at the site of carboxylation in modified bundle sheath cells. To investigate the feasibility of this, we generated a quadruple line that simultaneously accumulates four of the core C_4_ photosynthetic enzymes from the NADP-malic enzyme subtype, phospho*enol*pyruvate carboxylase (*Zm*PEPC), NADP-malate dehydrogenase (*Zm*NADP-MDH), NADP-malic enzyme (*Zm*NADP-ME), and pyruvate phosphate dikinase (*Zm*PPDK). This led to enhanced enzyme activity and mild phenotypic perturbations but was largely neutral in its effects on photosynthetic rate. Measurements of the flux of ^13^CO_2_ through photosynthetic metabolism revealed a significant increase in the incorporation of ^13^C into malate, consistent with increased fixation of ^13^CO_2_ via PEP carboxylase in lines expressing the maize PEPC enzyme. However, there was no significant differences in labeling of 3-phosphoglycerate (3PGA) indicating that there was no carbon flux through NADP-ME into the Calvin-Benson cycle. There was also no significant difference in labeling of phospho*enol*pyruvate (PEP) indicating that there was no carbon flux through PPDK. Crossing the quadruple line with a line with reduced glycine decarboxylase H-protein (*Os*GDCH) abundance led to a photosynthetic phenotype characteristic of the reduced *Os*GDCH line and higher labeling of malate, aspartate and citrate than in the quintuple line. There was evidence of ^13^C labeling of aspartate indicating ^13^CO_2_ fixation into oxaloacetate by PEPC and conversion to aspartate by the endogenous aspartate aminotransferase activity. While Kranz anatomy or other anatomical modifications have not yet been installed in these plants to enable a fully functional C_4_ cycle, these results demonstrate for the first-time a partial flux through the carboxylation phase of NADP-ME C_4_ metabolism in transgenic rice containing two of the key metabolic steps in the C_4_ pathway.

## Introduction

A major recent research objective has been the engineering of a C_4_ photosynthetic pathway into rice^1^ (Kajala et al., 2011; von Caemmerer et al., 2012; Ermakova et al., 2019), potentially leading to an increase in radiation use efficiency and yield of up to 50% (Hibberd et al., 2008). The C_4_ pathway represents a complex combination of both biochemical and anatomical adaptations that suppresses photorespiration by effectively saturating ribulose bisphosphate carboxylase/oxygenase (Rubisco) with CO_2_. In the majority of C_4_ plants, this is achieved by compartmentalization of photosynthetic reactions between two morphologically distinct cell types: the mesophyll cells (MCs) and the bundle sheath cells (BSCs). Operating across these cells is a biochemical CO_2_ pump elevating the CO_2_ concentration in the BSCs where Rubisco is located (Hatch, 1987).

There are three primary variants of this pump characterized by the main decarboxylase reaction (Hatch et al., 1975). The NADP-ME subtype was chosen for engineering C_4_ photosynthesis into rice as it is well-characterized in the C_4_ model crop species maize (*Zea mays*) and potentially requires the fewest biochemical enzymes among all C_4_ subtypes (Weber and von Caemmerer, 2010; Kajala et al., 2011; Ermakova et al., 2019). Each molecule of CO_2_ entering the cytosol of the MCs is first converted to bicarbonate (HCO_3_^–^) by the activity of carbonic anhydrase (CA) and then incorporated into phospho*enol*pyruvate (PEP) by PEP carboxylase (PEPC), yielding the C_4_ acid oxaloacetate (OAA). OAA is taken up into the chloroplast of the MCs where it is reduced to malate by the NADP-dependent malate dehydrogenase (NADP-MDH). Malate is exported back to the cytosol and then diffuses into BSCs through plasmodesmata along a steep concentration gradient. In the BSCs, malate is transported into the chloroplast by an unknown transporter and oxidatively decarboxylated by NADP-dependent malic enzyme (NADP-ME), yielding CO_2_, NADPH, and pyruvate. CO_2_ is assimilated by Rubisco, yielding two molecules of 3PGA, about half of which is reduced to triose-phosphate (TPs) using the NADPH provided by NADP-ME in the BSC chloroplast to regenerate RuBP in the Calvin-Benson cycle. The other half of the 3PGA moves to the MCs for reduction to TP in the MC chloroplast and then returns to the BSCs to enter the Calvin-Benson cycle. Pyruvate moves from the BSCs into the chloroplasts of the MCs where it is converted to PEP by pyruvate:phosphate dikinase (PPDK).

Previous attempts to introduce a single-cell C_4_ pathway into rice led to increased photoinhibition of photosynthesis, leaf chlorophyll bleaching and serious stunting with no evidence of CO_2_ concentration in chloroplasts (Taniguchi et al., 2008; Miyao et al., 2011). This work highlighted the need to achieve the correct activity, regulation, kinetic properties, and location of the enzymes. To address this, in this study, we report on the introduction of part of two-celled biochemical pathway into rice. It has previously been shown that genomic sequences encoding C_4_ proteins give stronger expression in rice than cDNAs (Matsuoka et al., 1994). Therefore, we decided to express individual full-length genes of *Zm*PEPC, *Zm*PPDK, *Zm*NADP-MDH, and *Zm*NADP-ME (including promoters, untranslated regions, exons, and introns) from maize (Kajala et al., 2011; Karki et al., 2020) in a bid to achieve a C_4_-like pattern of C_4_ gene expression, enzyme localization, enzyme activity, and enzyme kinetic properties (Matsuoka et al., 1994; Miyao, 2003; Hibberd and Covshoff, 2010; Miyao et al., 2011). Individual lines were then crossed to generate a plant overexpressing all four of these core C_4_ cycle enzymes to investigate the feasibility of installing a functional C_4_ biochemical pathway into rice. This quadruple transgenic line was also crossed with a line with decreased *Os*GDCH protein (Lin et al., 2016). We investigated the effect on plant growth and photosynthesis.

To evaluate photosynthetic functionality, we used ^13^CO_2_ labeling experiments (Arrivault et al., 2017), similar in concept to the radiolabeling experiments originally performed to characterize flux in C_4_ photosynthesis (Hatch et al., 1967; Hatch, 1971). Flux of ^13^CO_2_ through photosynthetic metabolism, in particular into C_4_ acids, was determined for the quadruple and quintuple lines, compared to untransformed controls. We show that there was increased labeling of C_4_ acids in both sets of plants compared to wild type, consistent with partial low-level function of a portion of the C_4_ pathway.

## Materials and Methods

### Plant Materials

Individual transgenic lines were generated overexpressing four of the core C_4_ cycle enzymes required for a functional NADP-ME C_4_ cycle (Kajala et al., 2011; Supplementary Figure 1), *Zm*PEPC (GRMZM2G083841), *Zm*PPDK (GRMZM2G306345), *Zm*NADP*-*MDH (GRMZM2G129513), and *Zm*NADP-ME (GRMZM2G085019. Generations of pSC0/*Zm*PEPC, pSC0/*Zm*PPDK, pSC0/ZmNADP-MDH, and pSC0/NADP-ME vectors were previously described (Giuliani et al., 2019b; Karki et al., 2020). In almost all cases, three independent single insertion homozygous transgenic lines with high transgene expression were selected for molecular and biochemical evaluation. However, for *Zm*NADP-ME, protein expression was only detected in a single transgenic line containing >6 copies of the overexpression construct and so this was the only line that could be taken forward (Karki et al., 2020). The overexpression constructs were stacked into single lines through conventional crossing to create two triple cross line (PEPC-28/PPDK-11/MDH-40 and PEPC-62/PPDK-2/MDH-22) each with an independent transgenic event for each gene. The crossing strategy was presented in Supplementary Figure 2. The *Zm*PEPC (PEPC-28 and PEPC-62) and *Zm*NADP*-*MDH (MDH-40 and MDH-22) single transgenic lines were initially crossed to create two double transgene lines (PEPC-28/MDH-40 and PEPC-62/MDH-22) that were then crossed with the *Zm*PPDK (PPDK-11 and PPDK-2) single transgenic lines. These two triple lines (PEPC-28/PPDK-11/MDH-40 and PEPC-62/PPDK-2/MDH-22) were then crossed with the single *Zm*NADP*-*ME (ME-116) transgenic line to produce two quadruple lines (PEPC-28/PPDK-11/MDH-40/ME-116 and PEPC-62/PPDK-2/MDH-22/ME-116). Progeny of the PEPC-28/PPDK-11/MDH-40 showed that detectable *Zm*PEPC localizes to MCs, but progeny of the PEPC-62/PPDK-2/MDH-22 showed that *Zm*PEPC localizes to both MCs and BSCs (Supplementary Figure 3). The PEPC-28/PPDK-11/MDH-40/ME-116 line was selected for analysis in the present study since its parent PEPC-28/PPDK-11/MDH-40 showed that correct *Zm*PEPC MCs expression and its progenitor PEPC-28 had been detail characterized in Giuliani et al. (2019b). Two quintuple crosses (PEPC-28/PPDK-11/MDH-40/ME-116/*gdch*-31) and (PEPC-28/PPDK-11/MDH-40/ME-116/*gdch*-38) were then generated by crossing the quadruple F_2_ line (PEPC-28/PPDK-11/MDH-40/ME-116) with single *Osgdch* knockdown lines (*gdch*-31 and *gdch*-38) described by Lin et al. (2016). The PEPC-28/PPDK-11/MDH-40/ME-116/*gdch*-38 line was chosen for analysis in present study since its progenitor *gdch*-38 had shown a more consistent photorespiratory-deficient phenotype under different O_2_:CO_2_ growing and measuring conditions compared with *gdch*-31 line, and had been detail characterized in Giuliani et al. (2019a). The presence of transgenes was determined by genomic PCR and protein accumulation by immunoblotting in each crossed line (Supplementary Figures 4–6).

### Plant Growth

Plants were grown under natural light conditions in a screenhouse with a day/night temperature of 35/28 ± 3°C at the International Rice Research Institute (Los Baños, Philippines: 14° 10019.900N, 121° 15022.300E). Maximum irradiance was 2000 μmol photons m^–2^ s^–1^ on a sunny day. Plants were grown in 7-liter pots filled with soil from the IRRI upland farm.

### Immunoblotting

Leaf samples for soluble protein extraction were harvested from the youngest fully expanded leaf at the mid-tillering stage between 09:00 h and 11:00 h, and stored on ice immediately. Leaves were homogenized to a fine powder using a nitrogen-cooled mortar and pestle. Proteins were extracted and fractionated by SDS-PAGE as described previously (Lin et al., 2016). Samples were loaded based on equal leaf area (0.2364 mm^2^ for *Zm*PEPC and *Zm*PPDK, and 2.364 mm^2^ for *Zm*NADP*-*MDH, *Zm*NADP*-*ME and *Os*GDCH). After electrophoresis, proteins were electroblotted onto a polyvinylidene difluoride membrane and probed with rabbit antisera against *Zm*PEPC, *Zm*NADP*-*MDH, *Zm*NADP*-*ME (all provided by Richard Leegood, University of Sheffield, United Kingdom), *Zm*PPDK (provided by Chris Chastain, Minnesota State University, United States), and *Os*GDCH protein (provided by Asaph Cousins, Washington State University, United States). The dilutions of *Zm*PEPC, *Zm*PPDK, *Zm*NADP*-*MDH, *Zm*NADP*-*ME, and *Os*GDCH antisera were 1:20,000, 1:20,000, 1:5,000, 1:2,000, and 1:100, respectively. A peroxidase-conjugated goat anti-rabbit IgG secondary antibody (Sigma-Aldrich, United States)^2^ was used at a dilution of 1:5,000 and immunoreactive bands were visualized with ECL Western Blotting Detection Reagents (GE Healthcare, United Kingdom)^3^.

### Immunolocalization

The middle portion of the youngest fully expanded leaf at the mid-tillering stage was sampled between 09:00 h and 11:00 h and processed as described previously by Lin et al. (2016). After fixation and cutting, the thin leaf sections were probed with the antisera against *Zm*NADP*-*MDH, *Zm*NADP*-*ME, *Zm*PPDK, and *Zm*PEPC at dilutions of 1:500, 1:25, 1:10, and 1:200, respectively. The secondary Alexa Fluor 488 goat anti-rabbit IgG (Invitrogen, United States)^4^ antibody was used at a dilution of 1:200. The sections were visualized on a BX61 microscope fitted with a Disk Scanning Unit attachment microscope (Olympus, United States)^5^ with fluorescence function under DAPI, RFP, and GFP filters.

### Enzyme Activity Measurement

Leaf samples were harvested between 09:00 h and 11:00 h from the youngest fully expanded leaf of plants at the mid-tillering stage, and frozen immediately. Leaves were homogenized to a fine powder using a nitrogen-cooled mortar and pestle and extracted in 250 μL of buffer containing: 50 mM HEPES-KOH, pH 7.4, 5 mM MgCl_2_, 1 mM EDTA, 1 mM Dithiothreitol, 1% (v/v) glycerol. After centrifugation at 10,000 × *g* for 2 min at 4°C, the supernatant was collected for enzyme activity measurements. PEPC enzyme activity was assayed using a method modified from Meyer et al. (1988) and Ueno et al. (1997). The PEPC reaction mixture contained: 100 mM HEPES-NaOH, pH 7.5, 10 mM MgCl_2_, 1 mM NaHCO_3_, 5 mM G6P, 0.2 mM NADH, 12 unit/mL MDH (from pig heart; Roche Diagnostics, Basel, Switzerland)^6^, and the reaction was started by adding PEP to a final concentration of 4 mM. PPDK enzyme activity was assayed as described by Fukayama et al. (2001). NADP-MDH activity was determined by a method modified from Tsuchida et al. (2001). NADP-MDH reaction mixture contained: 50 mM HEPES-KOH, pH 8, 70 mM KCl, 1 mM EDTA, 1 mM DTT, and 0.2 mM NADPH, and the reaction was started by adding OAA to a final concentration of 1 mM. NADP-ME activity was measured by a method modified from Tsuchida et al. (2001; protocol 1). The activities of PEPC, PPDK, NADP-MDH, and NADP-ME were measured spectrophotometrically at 340 nm at 25°C, 30°C, 25°C, and 25°C, respectively.

### Gas Exchange Measurements

Leaf gas-exchange measurements were made using a Li-6400XT infrared gas analyzer (LI-COR Biosciences, United States)^7^ fitted with a standard 2 × 3 cm leaf chamber and a 6400-02B light source. Measurements were made at a constant airflow rate of 400 μmol s^–1^, leaf temperature of 25°C, leaf-to-air vapor pressure deficit between 1.0 and 1.5 kPa and relative humidity of 60–65%. Data were acquired between 08:00 h and 13:00 h. Measurements were made from the two youngest fully expanded leaves for each plant during the tillering stage. The mid-portions of leaves were acclimated in the cuvette for approximately 30 min before measurements were made. The response curves of the net CO_2_ assimilation rate (*A*, μmol m^–2^ s^–1^) to changing intercellular *p*CO_2_ concentration (*C*_*i*_, μmol CO_2_ mol air^–1^) were acquired by decreasing *C*_*a*_ (*p*CO_2_ concentration in the cuvette) from 2000 down to 20 μmol CO_2_ mol air^–1^ at a photosynthetic photon flux density (PPFD) of 1500 or 2000 μmol photon m^–2^ s^–1^. The CO_2_ compensation point (*Γ*) and maximum carboxylation efficiency (*CE*) were calculated from the intercept (Vogan et al., 2007) and slope (Wang et al., 2006) of the CO_2_ response curves. Light response curves were acquired by increasing the PPFD from 0 to 2000 μmol photon m^–2^ s^–1^ at *C*_*a*_ 400 μbar. The quantum efficiency for CO_2_ assimilation (φ) and respiration rates (*R*_*d*_) were calculated from the slope and intercept of the light-response curves (PPFD < 100 μmol photons m^–2^ s^–1^).

### ^13^CO_2_ Pulse-Labeling and Quenching Procedure

Carbon flux analysis was performed with a custom-built gas exchange freeze clamp apparatus (Supplementary Figure 7). Measurements were made from two youngest fully expanded leaves for each plant during the tillering stage. Two leaves of up to 22 cm in length were placed inside a gas exchange chamber (23.5 cm × 4.5 cm × 0.4 cm), the top was constructed from a piece of clear flexible plastic to allow light penetration and the bottom from a sheet of aluminum foil to accelerate cooling when freeze clamping. The foil and plastic were attached with foil tape to a three-sided aluminum frame to provide rigidity. Two holes were drilled through the side of the frame to accommodate the air inlet and outlet tubes. A third hole on the end enabled thermocouples to be threaded through the frame to measure leaf and air temperature inside the labeling chamber. The chamber was then placed in a mounting frame allowing the leaf to be inserted prior to sealing the chamber with a foam gasket secured with bulldog clips. The mounting frame was positioned horizontally between two LED banks capable of providing illumination to the upper leaf surface of up to 1,000 μmol photons m^2^ s^–1^.

Air was drawn from outside the laboratory through a compressor. The air stream passed through an oil water separator and flow control valve into a copper coil placed in an ice bath for cooling. The air stream could be directed into the leaf chamber or by-passed into a CO_2_ conditioning unit. In the latter, CO_2_ could be removed from the air with soda lime and then optionally enriched with ^13^CO_2_ gas (300 ppm) prepared by mixing NaH^13^CO_3_ (Sigma-Aldrich, United States; see text footnote 2) with 2-hydroxypropanoic acid (lactic acid). The flow of air passing over the leaf (3 ml/min) was adjusted with a flow controller, and the CO_2_ concentration of the incoming and outgoing air streams measured with two CO_2_ analyzers (WMA-5, PP-Systems, United States)^8^. The humidity of the air inside the chamber was maintained at ∼60% with the addition of water to the soda lime chamber or reduced by passing air through a chamber of silica gel.

The leaf chamber was mounted on the stand in such a way that the plane of the leaf was halfway between two pneumatically operated aluminum bars. These were cooled with liquid nitrogen, and when released they clamped together fitting inside the aluminum chamber frame, very rapidly freezing the leaf. A fan was mounted horizontally to the bars to blow the fog from the liquid nitrogen away from the chamber to ensure there was minimal disruption to the environment before freezing occurred. The lower bar was positioned in such a way as to push the chamber up on closure. The cold temperatures and force of the bars closing meant the chamber disintegrated enabling the leaf to be removed with tweezers and placed in a liquid nitrogen bath for 10 s before subsequent storage at −80°C. To perform ^13^CO_2_ pulse-labeling, leaves were acclimated at steady-state conditions prior to scrubbing CO_2_ from the incoming air stream, then subjected to ^13^CO_2_ enriched air for a duration of 0 and 60 s and metabolic activity was quenched at these time points by freeze clamping the leaves as above. Freeze-quenched tissue was ground into a fine powder by mortar and pestle in liquid nitrogen. Finely ground leaf tissues were freeze-dried for 3 days and placed into sealed tubes. The sealed tubes containing finely ground lyophilized leaf tissue samples were shipped to Max Planck Institute of Molecular Plant Physiology, Germany for the metabolite analyses.

### Metabolite Analyses and Calculation of Total Pool Size, Enrichment and Isotopomer Distribution

Aliquots (3 or 5 mg) of finely ground lyophilized rice leaf tissue were extracted with chloroform-methanol as described in Arrivault et al. (2017), and the lyophilized extracts were resuspended in 300 μL or 600 μL purified (Millipore, United States) water, respectively. Isotopomers were measured by reverse-phase LC-MS/MS (malate, aspartate, 3PGA, PEP, citrate + isocitrate; Arrivault et al., 2017; n.b. citrate and isocitrate were not resolved using this method) and anion-exchange LC-MS/MS (malate, PEP, citrate; Lunn et al., 2006 with modifications as described in Figueroa et al., 2016). Total amounts of malate, aspartate, citrate + isocitrate, and citrate were calculated by summing isotopomers. The total amounts of 3PGA and PEP were determined enzymatically in trichloroacetic acid extracts using a Sigma-22 dual-wavelength photometer (Merlo et al., 1993), with PEP being measured in freshly prepared extracts. ^13^C enrichment and relative isotopomer distribution were calculated as in Szecowka et al. (2013).

### Statistical Analysis

Statistical analysis for all experiments was performed in R version 3.0.0 (The R Foundation for Statistical Computing, Vienna, Austria) using a one-way analysis of variance (ANOVA) and a Tukey *post hoc* test or a Student’s *t*-test with a *p*-value of <0.05.

## Results

### Overexpression of C_4_ Cycle Genes in *Oryza sativa*

**FIGURE 1:**
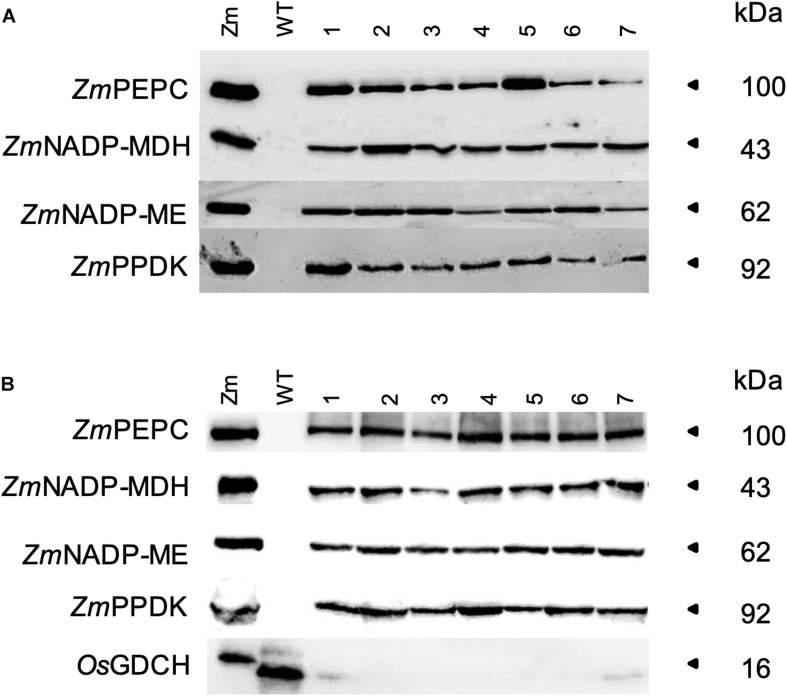
Soluble leaf protein. Immunoblots for **(A)** Quadruple and **(B)** Quintuple crosses. Maize (Zm), wild-type rice (WT), and plants of F_2_ crosses (numbers). Protein was extracted from the youngest fully expanded leaf at mid-tillering stage. Samples were loaded on an equal leaf area basis (0.2364 mm^2^ for *Zm*PEPC and *Zm*PPDK, and 2.364 mm^2^ for *Zm*NADP-MDH, *Zm*NADP-ME, and *Os*GDCH).

Immunoblotting of F_2_ generation transgenic plants showed that the protein of the correct size for *Zm*PEPC, *Zm*NADP-MDH, *Zm*NADP-ME, and *Zm*PPDK was stably expressed in both the quadruple and quintuple cross rice lines, with *Os*GDCH protein almost undetectable in the quintuple cross (Figure 1). In all lines, protein abundance was lower than that of wild-type maize plants. We next sought to determine whether these proteins were localized to the correct cell type and subcellular compartment. Immunolocalization analysis of the quadruple cross line revealed that *Zm*PEPC was localized to the cytosol of MCs similar to the single *Zm*PEPC transgenic line (Giuliani et al., 2019b) and the triple cross line (Supplementary Figure 3). *Zm*PPDK, was localized to the chloroplast in both BSCs and MCs (Supplementary Figure 8). The *Zm*NADP-MDH and *Zm*NADP-ME antisera cross-reacted with native protein in wild-type rice and so it was not possible to distinguish protein encoded by the endogenous rice gene from that encoded by the maize transgene. Overexpression of these enzymes conferred enhanced enzyme activity (Supplementary Table 1). In the quadruple cross line, PEPC activity was 18.4-fold higher compared to wild-type rice plants, PPDK 5.8-fold higher, NADP-MDH 9.9-fold, and NADP-ME 4.1-fold (Table 1).

**TABLE 1:** The enzyme activities of PEPC, NADP-MDH, NADP-ME, and PPDK.

	PEPC	NADP-MDH	NADP-ME	PPDK
	μmol s^–1^ m^–2^	μmol s^–1^ m^–2^	μmol s^–1^ m^–2^	μmol s^–1^ m^–2^
Maize	42.54 ± 1.09^*a*^	12.81 ± 0.39^*b*^	24.21 ± 2.38^*a*^	9.44 ± 1.34^*a*^
WT	1.03 ± 0.01^*c*^	3.50 ± 0.03^*c*^	0.56 ± 0.07^*c*^	0.30 ± 0.03^*c*^
Quadruple	18.96 ± 2.56^*b*^	34.98 ± 3.26^*a*^	2.34 ± 0.10^*b*^	1.76 ± 0.14^*b*^

*Values are average ± SE of 2–3 plants of maize, wild-type rice (WT), and F_2_ quadruple crosses. Different letters within groups indicated those values that are statistically different based on a one-way ANOVA, P-value < 0.05.*

### Phenotypic and Photosynthetic Perturbations Associated With Overexpression of C_4_ Cycle Genes

**FIGURE 2:**
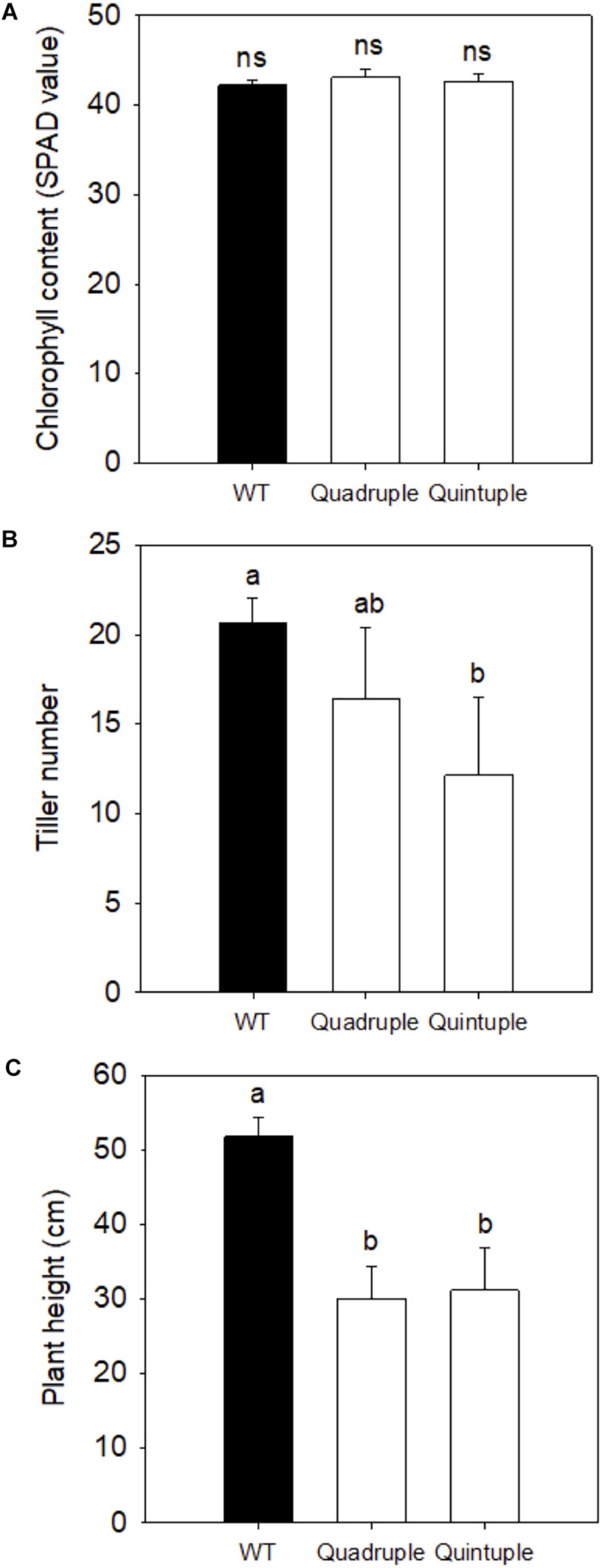
**(A)** Leaf Chlorophyll content, **(B)** tiller number, and **(C)** plant height of wild-type (WT), Quadruple, and Quintuple lines. Chlorophyll SPAD values, tiller number, and plant height are means ± SE of eight individual F_2_ plants and eight WT plants, 90 days post germination. Different letters within groups indicated those values that are statistically different based on a one-way ANOVA with a Tukey multiple comparison test for *post hoc* pairwise comparison, *P*-value < 0.05. ns indicates non-significant.

Given that protein levels and activities of all four introduced C_4_ enzymes were enhanced compared to wild-type plants, we investigated whether this affected growth and photosynthesis. None of the crossed lines consistently showed altered chlorophyll content (Figure 2A). Tiller number in the quintuple cross lines (Figure 2B) and plant height in both quadruple and quintuple crosses (Figure 2C) were significantly reduced. Phenotypic perturbations were most marked in the quintuple cross lines (Supplementary Figure 9), although the plants still developed and flowered at the same time with wild-type plants. These growth perturbations were not observed in the single C_4_ gene transgenic lines of *Zm*PEPC, *Zm*PPDK, *Zm*NADP*-*MDH, and *Zm*NADP*-*ME (Giuliani et al., 2019b) but were observed in the single *Osgdch* knockdown line (Lin et al., 2016).

**FIGURE 3:**
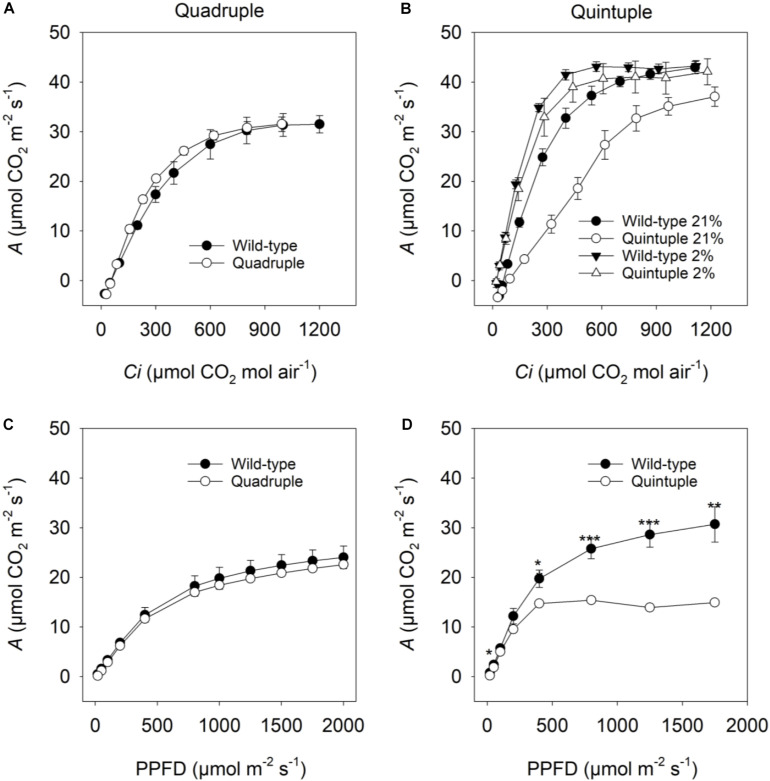
**(A,B)** Net CO_2_ assimilation rate (*A*) in response to intercellular *p*CO_2_ (*Ci*) and **(C,D)** photosynthetic photon flux density (PPFD). Measurements **(A,B)** were made at 2000 μmol photons m^–2^ s^–1^ under 21% O_2_ **(A,B)** and 2% O_2_ **(B)**. Measurements **(C,D)** were made at a *p*CO_2_ (*C*_*a*_) of 400 μmol CO_2_ mol air^–1^. Values are means ± SE of three F_2_ cross plants and three wild-type (WT) plants for quadruple comparisons and seven F_2_ cross plants and four WT plants for quintuple comparisons. A Student’s *t*-test was performed for **(C,D)**. Significant differences between WT and quintuple within PARi level are indicated by *P-value < 0.05, **P-value < 0.01, ***P-value < 0.001.

To investigate whether overexpression of C_4_ genes impacted photosynthesis, the response of net CO_2_ assimilation rate (*A*) to CO_2_ concentration under photorespiratory conditions (21% O_2_) was measured. In the quadruple cross line there were no differences in CO_2_ assimilation (Figures 3A,C), *Γ*, CE, *R*_*d*_ or Φ compared to wild-type plants (Table 2). These results suggested that accumulation of active maize C_4_ cycle enzymes in rice does not significantly affect leaf level photosynthetic gas-exchange and that the phenotypic perturbations are not associated with reduced CO_2_ assimilation.

**TABLE 2:** Comparison of photosynthetic parameters.

	Γ	*CE*	R_*d*_	Φ
	μmol CO_2_ m^–2^ s^–1^	μmol CO_2_ m^–2^ s^–1^ μmol CO_2_ mol^–1^	μmol CO_2_ m^–2^ s^–1^	mol CO_2_ mol^–1^ quanta
WT 21% O_2_	53.44 ± 0.40	0.11 ± 0.01	0.23 ± 0.04	0.04 ± 0.00
Quadruple 21% O_2_	56.11 ± 5.24	0.10 ± 0.01	0.55 ± 0.25	0.03 ± 0.00
WT 21% O_2_	59.48 ± 1.70^*b*^	0.13 ± 0.01^*a*^	0.60 ± 0.20^*b*^	0.06 ± 0.01^*a*^
Quintuple 21% O_2_	88.76 ± 4.13^*a*^	0.05 ± 0.00^*b*^	1.09 ± 0.23^*a*^	0.05 ± 0.00^*b*^
WT 2% O_2_	23.17 ± 5.07	0.19 ± 0.01^*a*^	–	–
Quintuple 2% O_2_	21.34 ± 1.74	0.15 ± 0.01^*b*^		–

*CO_2_ compensation point (Γ), carboxylation efficiency (CE), respiration rates (R_d_), and quantum yield for CO_2_ assimilation (φ). Measurements of Γ and CE were made at a PPFD of 2000 μmol photons m^–2^ s^–1^. Φ at a pCO_2_ (C_a_) of 400 μmol CO_2_ mol air^–1^ and a leaf temperature of 25°C. Values are means ± SE of three F_2_ cross plants and three wild-type (WT) plants for quadruple comparisons and seven F_2_ cross plants and four WT plants for quintuple comparisons. Different letters within groups indicated those values that are statistically different based on a Student’s t-test, P-value < 0.05.*

For the quintuple cross, the CO_2_ assimilation rate in response to increasing intercellular CO_2_ concentrations (*Ci*) was reduced, most notably at lower CO_2_ concentrations (<700 μmol CO_2_ mol^–1^, Figure 3B). Under non-photorespiratory conditions (2% O_2_, Figure 3B) CO_2_ assimilation rates were similar to wild-type plants. Consistent with this, the quintuple cross had a significantly higher *Γ* under high photorespiratory conditions but not under low photorespiratory conditions (Table 2). In response to changes in photon flux density, photosynthesis was saturated at lower light levels than in wild-type plants (400 μmol photons m^–2^ s^–1^ versus 1750 μmol photons m^–2^ s^–1^, respectively, Figure 3D) with significantly lower Φ and higher *R*_*d*_ than wild-type plants (Table 2).

To investigate these photosynthetic responses for the quintuple cross line in more detail, CO_2_ responses were measured under conditions conducive to low and high rates of photorespiration. Under low light (400 μmol photons m^–2^ s^–1^) and high CO_2_ (2000 μmol CO_2_ mol air^–1^), conditions conducive to low photorespiration, CO_2_ responses of the quintuple cross line were similar to wild-type plants (Supplementary Figures 10A,D). Under high light (1500 μmol photons m^–2^ s^–1^) and low CO_2_ (400 μmol CO_2_ mol air^–1^), conditions conducive to high photorespiration, CO_2_ assimilation was lower in the quintuple cross (Supplementary Figures 10B,C). Correspondingly, Γ was higher, and carboxylation efficiency (*CE*) lower under conditions conductive to high rates of photorespiration but not under non-photorespiratory conditions (Supplementary Table 1). This is consistent with the photorespiratory-deficient phenotype observed in the single *Osgdch* knockdown lines (Lin et al., 2016).

### Increased Incorporation of ^13^C Into C_4_ Acids Associated With Overexpression of C_4_ Cycle Enzymes

**FIGURE 4:**
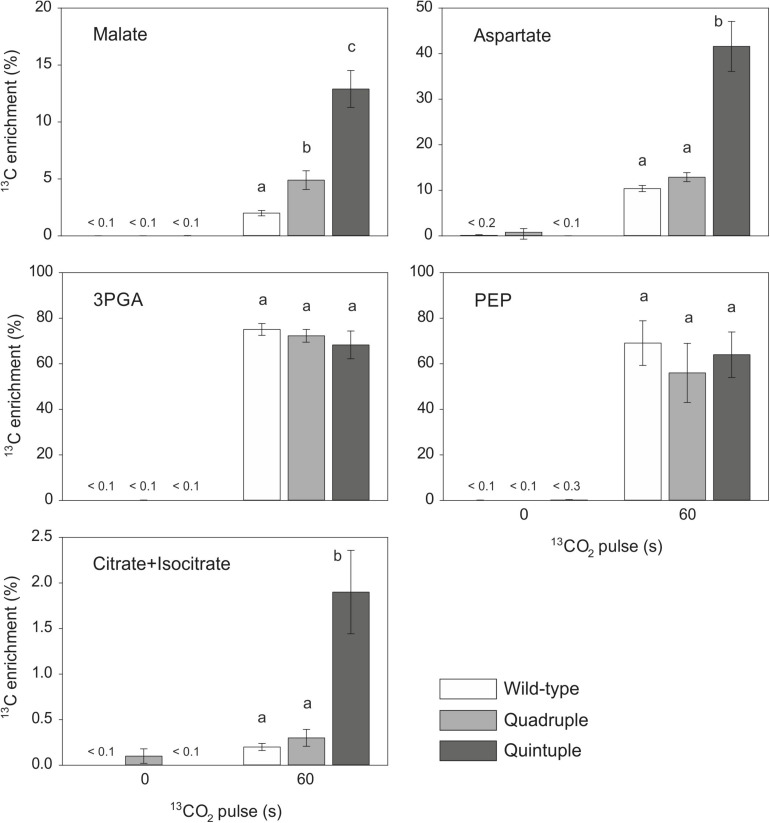
^13^CO_2_ pulse-labeling of wild-type, quadruple and quintuple rice cross lines. Fully expanded leaves at mid-tillering stage were pulse-labeled with ^13^CO_2_ (300 ppm) for 60 s under steady state photosynthetic conditions. Isotopomers of malate, aspartate, 3PGA, PEP, and citrate + isocitrate were measured in extracts from pulse-labeled (60 s) and non-labeled (0 s) leaves by LC-MS/MS, and ^13^C enrichment (%) was calculated after correction for natural abundance. Values are means ± SE (*n* = 2–4). The original data are presented in Supplementary Dataset A. Different letters within groups indicated those values that are statistically different based on a one-way ANOVA, *P*-value < 0.05.

We performed experiments to measure the flux of ^13^CO_2_ through photosynthetic metabolism to investigate whether there was partial functionality of a C_4_ pathway in these plants. There was significantly more incorporation of ^13^C into malate in the quadruple and quintuple cross lines than in wild-type plants (Figure 4 and Supplementary Figure 11), with the *m*_1_ isotopomer being more abundant than the other ^13^C-labeled isotopomers (Table 3), consistent with increased fixation of ^13^CO_2_ via PEPC in the transgenic lines expressing the maize PEPC enzyme. Labeling of aspartate was also significantly higher than wild-type in the quintuple line (Figure 4 and Supplementary Figure 11), with the *m*_1_ isotopomer being more abundant that *m_2_-m_4_* isotopomers (Table 3), indicating ^13^CO_2_ fixation into oxaloacetate by maize PEPC and conversion to aspartate by endogenous rice aspartate aminotransferase activity. There was no evidence of significant difference in labeling of aspartate in the quadruple line. There was almost complete labeling of the 3PGA pool in wild-type plants after a 60 s pulse, and similarly high levels of labeling of 3PGA were observed in the quadruple and quintuple lines (Figure 4 and Supplementary Figure 11). There was also substantial labeling of PEP after a 60 s pulse, with no significant differences between the transgenic lines and wild-type plants (Figure 4 and Supplementary Figure 11). There was almost no labeling of citrate and isocitrate in wild-type plants and the quadruple line (Figure 4). In contrast, the enrichment of ^13^C in citrate and isocitrate in the quintuple line was 10-fold higher than in wild-type plants.

**TABLE 3:** Relative isotopomer distribution (%) of malate and aspartate in wild-type, quadruple and quintuple rice lines after pulse-labeling with ^13^CO_2_ for 60 s.

		m_0_	m_1_	m_2_	m_3_	m_4_
Malate	Wild-type	96.4 ± 0.35	1.2 ± 0.17	1.0 ± 0.08	0.6 ± 0.07	0.7 ± 0.03
	Quadruple	90.5 ± 1.09	4.1 ± 0.64	2.4 ± 0.27	1.3 ± 0.12	1.6 ± 0.2
	Quintuple	79.3 ± 1.15	7.0 ± 0.02	3.3 ± 0.11	3.6 ± 0.19	6.8 ± 1.06
Aspartate	Wild-type	77.2 ± 0.19	11.6 ± 0.83	5.5 ± 0.29	3.8 ± 0.72	1.9 ± 0.01
	Quadruple	71.5 ± 1.67	14.7 ± 2.49	6.7 ± 1.15	4.7 ± 0.76	2.3 ± 1.22
	Quintuple	28.1 ± 7.37	26.6 ± 2.93	12.5 ± 2.13	16.4 ± 0.89	16.4 ± 1.41

*The relative abundance of each isotopomer (m_n_) for a given metabolite is represented; n is the number of ^13^C atoms incorporated. Values are means ± SE of 2–4 plants of wild-type rice and F_2_ quadruple crosses. The original data are presented in Supplementary Dataset A.*

## Discussion

We have previously shown that overproduction of individual C_4_ enzymes in rice has no consistent effect on CO_2_ assimilation or plant growth (Giuliani et al., 2019b; Karki et al., 2020). The exception to this was the transgenic line overexpressing *Zm*NADP-ME which exhibited a small decrease in plant height and reduced maximal photosynthetic rate at high CO_2_. Previous attempts to overproduce *Zm*NADP-ME in rice have led to increased photoinhibition of photosynthesis, leaf chlorophyll bleaching and serious stunting attributed to an increase in the NADPH/NADP^+^ ratio in the chloroplast stroma due to the exchange with 2-oxoglutarate involved in photorespiration (Tsuchida et al., 2001). Severe phenotypic effects were not observed in our *Zm*NADP-ME line; however, we were only able to advance a single line containing 6 copies of the construct in which protein accumulation was higher than in our rice control, but still only around 10% of the activity found in maize. In contrast, the experiments of Tsuchida et al. (2001) used a rice chlorophyll a/b binging protein (*cab*) promoter allowed for high level but not cell specific expression, and activities of up to 60% of maize levels were achieved, leading to a much more severe phenotype. Overproduction of all four targeted C_4_ enzymes in a single plant led to a slight decrease in tiller number and plant height but otherwise growth and photosynthesis were unaffected. These results are consistent with previous reports of engineering a single-cell C_4_ pathway in rice (Taniguchi et al., 2008). A quintuple cross that combined over-expression of the four C_4_ enzymes, *Zm*PEPC, *Zm*NADP-MDH, *Zm*NADP-ME, and *Zm*PPDK with knockdown of the native rice *Os*GDCH, thereby compromising the photorespiratory pathway, led to further reductions in tiller number and plant height. A strong negative effect on photosynthesis was also observed in the quintuple cross consistent with the photorespiratory-deficient phenotype of the single *Osgdch* knockdown line (Lin et al., 2016).

Our results show that *Zm*PEPC is catalytically active *in vivo* when expressed in combination with other C_4_ enzymes in rice, and substantially increases the fixation of CO_2_ into C_4_ acids. This is in contrast to published radiolabeling studies of rice expressing *Zm*PEPC alone (Fukayama et al., 2003; Miyao et al., 2011) in which there was no increase in incorporation of labeled carbon into C_4_ acids, despite the extractable activity of PEPC in these plants approaching or exceeding maize levels. Despite strong evidence for operation of a partial C_4_ pathway in our transgenic lines up to the point of malate production, there was no evidence for the regeneration of PEP via the rest of the C_4_ cycle. The labeling of PEP at a similar level in all three genotypes, wild-type, quadruple and quintuple crosses, suggests that rather than being produced by a functional C_4_ cycle, PEP is being produced from 3PGA via 2PGA catalyzed by phosphoglyceromutase and enolase (Furbank and Leegood, 1984), consistent with the majority of CO_2_ still being fixed via Rubisco in C_3_ photosynthesis rather than through the operation of a complete C_4_ cycle.

The very low incorporation of ^13^C into citrate and isocitrate in wild-type rice plants is consistent with previous studies (Tcherkez et al., 2009; Szecowka et al., 2013) indicating little flux of carbon into the tricarboxylic acid (TCA) cycle via mitochondrial pyruvate dehydrogenase (mPDH) in the light, due to deactivation of the mPDH by phosphorylation (Randall et al., 1996; Tovar-Méndez et al., 2003). The increased labeling of citrate in the quintuple cross line suggests that the mPDH is more active in the light in this line, potentially leading to respiration of C_4_ acids via the TCA cycle, which would be deleterious for C_4_ photosynthetic flux. This might be due to lower rates of photorespiration leading to less photophosphorylation of PDH (Tovar-Méndez et al., 2003). Further, increased levels of pyruvate in the mitochondria (from decarboxylation of malate by NAD-malic enzyme), can inhibit the mPDK kinase (Schuller and Randall, 1990). We propose that there is a modified regulation of the TCA cycle to avoid wasteful respiration of C_4_ acids, and that such modification might have been needed for the evolution of an efficient C_4_ photosynthetic pathway.

Evidence that *Zm*PEPC can be localized to the cytosol of MCs (Giuliani et al., 2019b) and is catalytically active, leading to the fixation of CO_2_ into C_4_ acids provides important evidence in support of installing a fully functional C_4_ photosynthetic pathway into rice. However, absolute quantification of flux into and through C_4_ acids would require further pulse-chase labeling studies and may prove difficult with the low rates of labeling relative to C_3_ photosynthetic fixation obtained in the current transgenic lines.

Achieving the correct cellular localization of the C_4_ enzymes introduced into the rice lines shown here remains an important and unresolved issue. We introduced intact maize C_4_-specific genes containing the promoter into rice (Karki et al., 2020) based on the strategy by Miyao et al. (2011). This approach was originally adopted because it had been reported that the 5′- flanking region of the maize C_4_ specific genes drove high-level MC-specific expression of a reporter β-glucuronidase (GUS) gene in rice leaves (Matsuoka et al., 1993, 1994). However, we knew at the time that this approach might not lead to cell specific expression of the enzymes (Sheen and Bogorad, 1987; Nomura et al., 2005). Miyao et al. (2011) had reported that expression of the native promoter and full-length maize PPDK gene in rice led to the accumulation of protein in both the MCs and BSCs. At the same time they also raised the possibility that maize PEPC might also accumulate in both cell types, Therefore, it was not unexpected that in the lines reported here, or the single transgenic lines used as parents for the crosses in this study (Karki et al., 2020), PPDK and PEPC accumulated in both cell types, It remains unclear why in selected events, maize PEPC appears to confined the BS (Giuliani et al., 2019b). Owing to the absence antibodies specific for *Zm*NADP-ME and *Zm*NADP-MDH, we have been unable to establish the cell localization of these enzymes. However, given that the promoters used, cell-specific expression seems unlikely (Nomura et al., 2005).

To mitigate the risk associated with this approach we also generated constructs where each coding sequence (CDS) was fused to either the M promoter *ZmPEPC* (Matsuoka et al., 1993) or the BS promoter *OsPCK1* (Nomura et al., 2005) with the *nopaline synthase* (*nos*) terminator at the 3′ end. These lines were not used as neither approach led to the cell specific expression (unpublished, W. P. Quick, personal communication). As no alternative strategy for achieving cell-specific expression was available at the time, lines containing the full length genes and native promoters were used as these lead to an enrichment of maize PEPC and PDDK in the correct cell type. Incorrect or partial localization of enzymes would potentially limit the operation of a C_4_ cycle (Miyao et al., 2011), with the potential to lead to deleterious phenotypes, i.e., (Tsuchida et al., 2001). Thus, the cell-specific expression of enzymes remains an active area of research for the consortium. It has been suggested that gene specificity may be generated by elements that are not present in the promoter (Hibberd and Covshoff, 2010).

In addition to high level, cell specific expression of C_4_ cycle enzymes in rice, fully functional C_4_ photosynthetic biochemistry requires appropriate enzyme regulation in the environment of a rice leaf cell (Burnell and Hatch, 1985; Chastain et al., 1997). For example, the activity of C_4_ specific PPDK is regulated in the light through protein phosphorylation by the PPDK regulatory protein (Burnell and Hatch, 1985). Similarly, NADP-MDH is regulated by light through the thioredoxin cascade (Miginiac-Maslow et al., 2000). Both enzymes are regulated in the same manner even when expressed within C_3_ leaves (Fukayama et al., 2001; Taniguchi et al., 2008). A recent study has shown that C_4_ NADP-ME is also regulated in the light by reversible phosphorylation at Ser419 which is involved in the binding of NADP at the active site (Bovdilova et al., 2019). In contrast, PEPC is regulated by both metabolite effectors and reversible phosphorylation, but the mechanisms of regulation in C_3_ and C_4_ leaves are different (Vidal and Chollet, 1997). Indeed, Fukayama et al. (2003) observed inappropriate phosphorylation of PEPC in their transgenic rice lines and proposed this as a reason for lack of labeling of C_4_ acids in the light. The regulatory mechanisms for other enzymes are less well understood. It is unclear at present whether enzyme levels *per se* or enzyme regulation in our rice transgenic lines, or both, is limiting C_4_ flux.

In NADP-ME C_4_ plants assimilation of a single CO_2_ molecule requires at least 10 transport steps between cells and within subcellular compartments of the MC and BSC. The identity of most of the transporter proteins supporting is now known, although there remains some uncertainty about malate import to the BS chloroplast and the export of pyruvate following malate decarboxylation (Weber and von Caemmerer, 2010; Ermakova et al., 2019). The next logical step is to introduce these into the current prototype. This was initially planned and the started as part of a 6-year strategy to develop a prototype expressing all known genes required to support the C_4_ biochemical pathway. Since then the emergence of Golden Gate cloning has enabled the consortium to reduce that strategy to 6-months by creating a large multigene overexpression construct (Ermakova et al., 2019). This makes the prototype development strategy adopted in this study obsolete.

A plethora of other changes are required to support a fully functional C_4_ pathway in rice. This includes, but is not limited to, engineering the correct leaf anatomy (Hattersley and Watson, 1975; Dengler et al., 1994; Dengler and Taylor, 2000; Muhaidat et al., 2007) and morphological specializations such as increased vein density (Sedelnikova et al., 2018). In addition, thought must be given to the photosynthetic functionalization of the BSCs of rice, which contain a large central vacuole, with very few mitochondria, peroxisomes or chloroplasts (Sage and Sage, 2009; Ermakova et al., 2019). Where chloroplasts do occur, they are smaller than those in MCs. Increasing chloroplast number and volume in the BSCs will no doubt be important for achieving C_4_ photosynthesis in rice (Chonan, 1970, 1978; Dengler et al., 1994; Ueno et al., 2006; Wang et al., 2017). Insufficient chloroplast volume in the BSCs of rice may have led to limitations in C_4_ acid decarboxylation in the transgenic lines described here. In addition, MCs of rice are highly lobed to assist with photorespiratory CO_2_ scavenging (Sage and Sage, 2009); whereas the MCs of C_4_ species are not. It is possible that these features may hinder the transport of metabolites between cells (Sage and Sage, 2009). Other modifications such as cross-sectional area of the BSCs, modifying the cell wall properties for diffusion of CO_2_ (von Caemmerer and Furbank, 2003) and increasing plasmodesmatal frequency at the BSC/MC interface to support metabolite diffusion may be necessary (Ermakova et al., 2019). The genetic regulators of many of these changes are not known, and so future goals include identification and incorporation of necessary genes for anatomical modifications into a version of the current biochemical prototype, with the ultimate goal of engineering an efficient C_4_ pathway in rice. The research presented here represents a small step toward this goal.

## Data Availability Statement

All datasets presented in this study are included in the article/Supplementary material.

## Author Contributions

HL, SA, RC, JL, MS, RF, and WQ designed the experiments together. HL provided all the plant materials. HL and EB performed enzyme activity assay, immunoblotting, immunolocalization, and gas exchange measurements. WQ, RF, MS, JL, and RC designed the gas exchange freeze clamp apparatus. SA performed metabolite analysis. HL, SA, RC, and WQ wrote the manuscript. SC and JH designed constructs. SK performed plant transformation. HL and RC performed the ^13^*CO*_2_ labeling experiment. All authors contributed to the article and approved the submitted version.

## Conflict of Interest

The authors declare that the research was conducted in the absence of any commercial or financial relationships that could be construed as a potential conflict of interest.

## Footnotes

1 https://C4rice.com

2 https://www.sigmaaldrich.com/

3 https://www.gelifesciences.com

4 https://www.thermofisher.com/ph/en/home/brands/invitrogen.html

5 https://www.olympus-global.com/

6 https://www.roche.com/

7 https://www.licor.com/

8 https://ppsystems.com/

## Supplementary Material

Supplementary Dataset A Isotopomer and metabolite amounts, ^13^C enrichments and relative isotopomer abundances of malate, aspartate, 3PGA, PEP and citrate+isocitrate in wild-type, quadruple and quintuple lines.

Supplementary Dataset B Isotopomer and metabolite amounts, ^13^C enrichments and relative isotopomer abundances of malate, aspartate, 3PGA, PEP, and citrate in wild-type and quintuple lines.
