# Supplementary material for: A Partial C4 Photosynthetic Biochemical Pathway in Rice
Source: Front Plant Sci. 2020 Oct 15;11:564463. doi: 10.3389/fpls.2020.564463 (PMC7593541; doi:10.3389/fpls.2020.564463)
Supplement: Supplementary file 2 [file Data_Sheet_1.PDF]

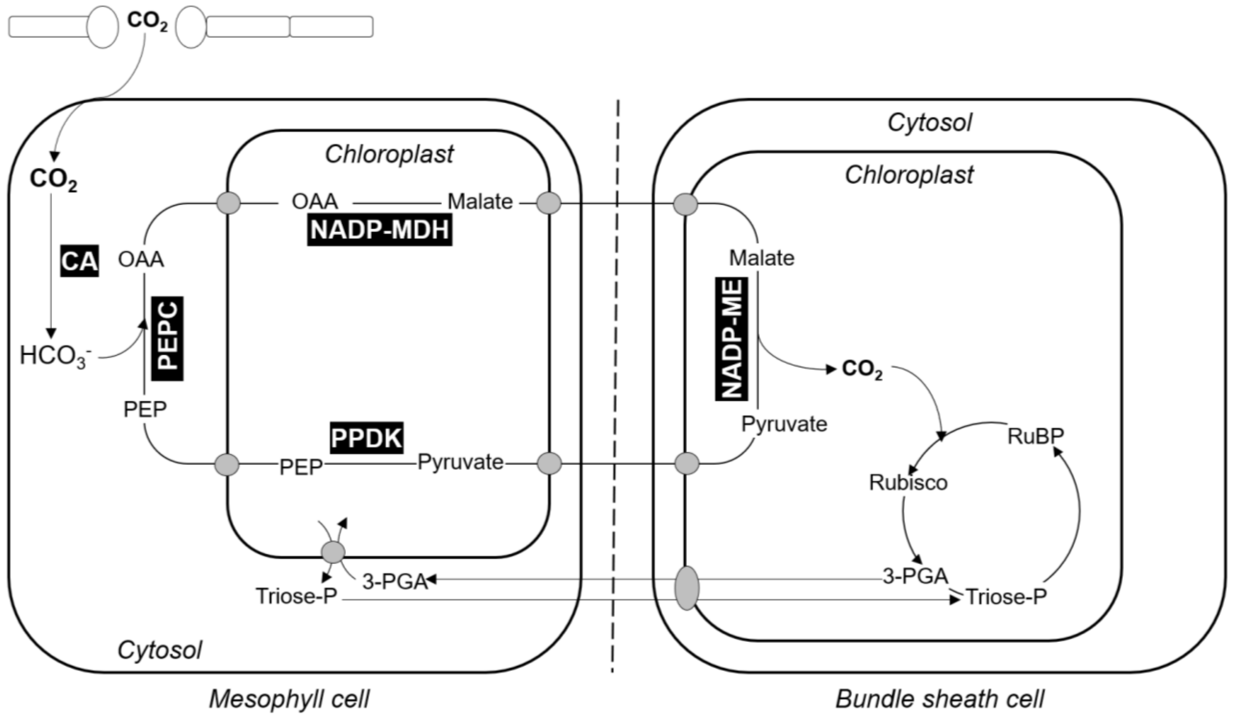

Supplementary Figure 1. Schematic representation of the NADP-ME subtype of C_4 photosynthesis. Carbon dioxide (CO_2) is converted to bicarbonate (HCO_3^-); this is then fixed by phospho*enol*pyruvate carboxylase (PEPC) catalyzing the formation of oxaloacetate (OAA). OAA is reduced to malate by NADP-dependent malate dehydrogenase (NADP-MDH). This is oxidatively decarboxylated by NADP-dependent malic enzyme (NADP-ME), yielding CO_2, NADPH and pyruvate. CO_2 is assimilated by Rubisco. Pyruvate is converted to PEP by pyruvate:phosphate dikinase (PPDK).

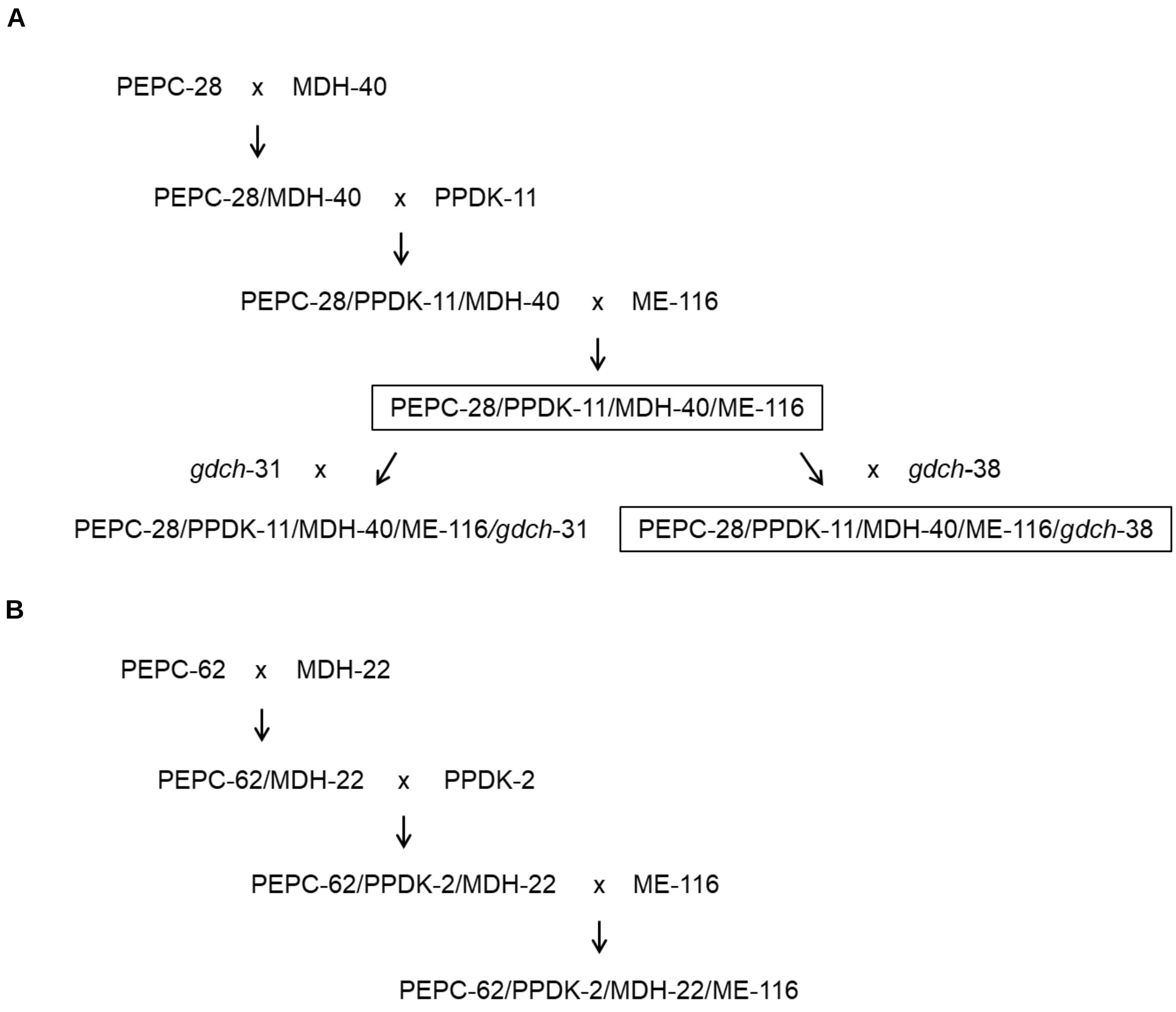

Supplementary Figure 2. Crossing strategy. **(A)** Pedigree of the triple line PEPC-28/PPDK-11/MDH-40, the quadruple line PEPC-28/PPDK-11/MDH-40/ME-116 and two quintuple lines, PEPC-28/PPDK-11/MDH-40/ME-116/*gdch*-31 and PEPC-28/PPDK-11/MDH-40/ME-116/*gdch*-38. **(B)** Pedigree of the triple line PEPC-62/PPDK-2/MDH-22 and the quadruple line PEPC-62/PPDK-2/MDH-22/ME-116.

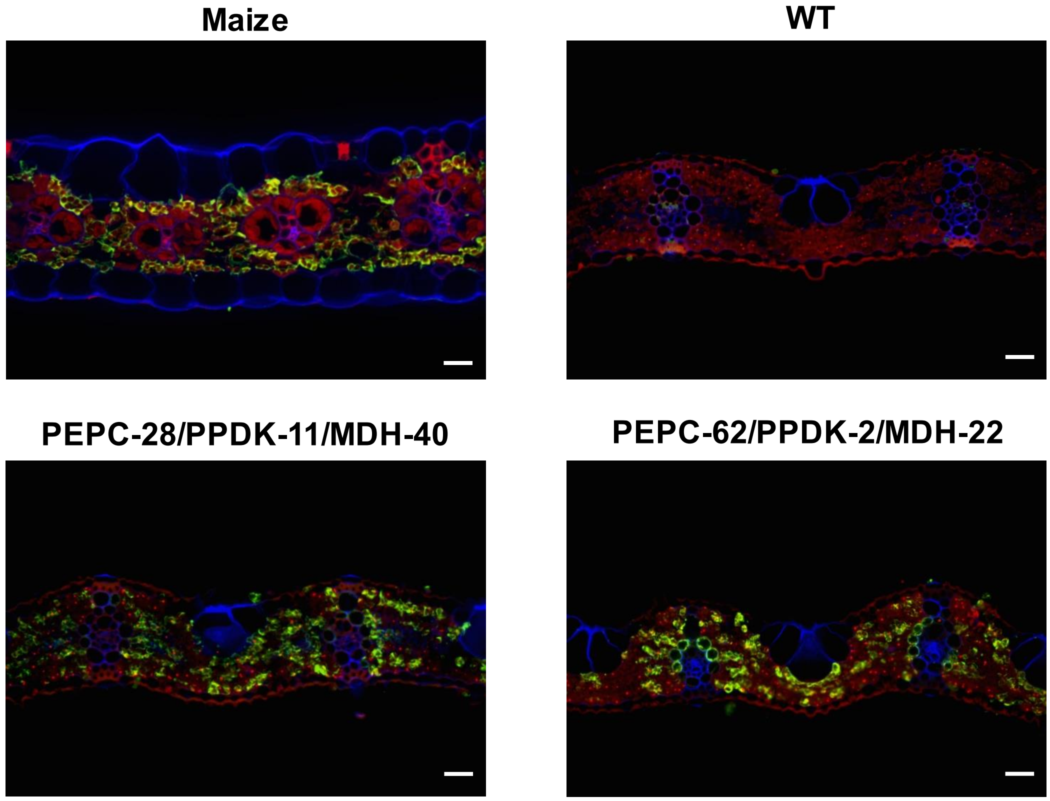

Supplementary Figure 3. Representative images of immunolocalization of *Zm*PEPC protein in triple crosses, PEPC-28/PPDK-11/MDH-40 and PEPC-62/PPDK-2/MDH-22. Anti-rabbit polyclonal primary antibodies (*Zm*PEPC 1:500) plus Alexa Fluor 488 goat anti-rabbit IgG as secondary antibody (1:200; shown in green color). Red shows autofluorescence of chlorophyll in chloroplast. Co-staining with calcofluor white visualized cell wall (shown in blue). Scale bar: 20 µm. Images are of the middle portion the seventh fully expanded fifth leaf of Maize, wild-type (WT) and F_1 generation triple crosses. Maize: positive control. Wild-type: negative control.

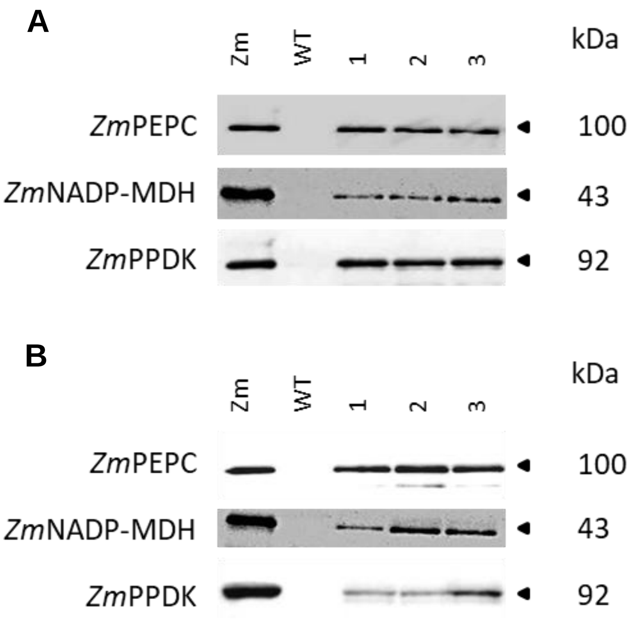

Supplementary Figure 4. Soluble leaf protein. Immunoblots for Triple crosses. **(A)** PEPC-28/PPDK-11/MDH-40; **(B)** PEPC-62/PPDK-2/MDH-22. Maize (Zm), wild-type rice (WT) and plants of F_1 crosses (numbers). Protein was extracted from the youngest fully expanded leaf at mid-tillering stage; samples were loaded on an equal leaf area (0.2364 mm^2 for *Zm*PEPC and *Zm*PPDK, and 2.364 mm^2 for *Zm*NADP-MDH).

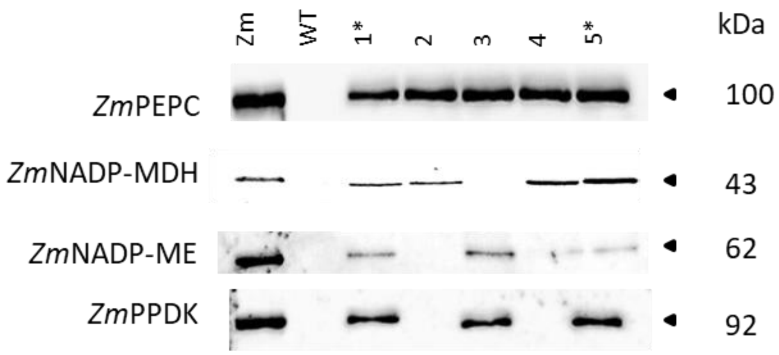

Supplementary Figure 5. Soluble leaf protein. Immunoblots for Quadruple (PEPC-62/PPDK-2/MDH-22/ME-116) crosses. Maize (Zm), wild-type rice (WT) and plants of F_1 crosses (numbers). Asterisks denote plants in which all four proteins (PEPC, PPDK, MDH and ME) were expressed. Protein was extracted from the youngest fully expanded leaf at mid-tillering stage; samples were loaded on an equal leaf area (0.2364 mm^2 for *Zm*PEPC and *Zm*PPDK, and 2.364 mm^2 for *Zm*NADP-MDH and *Zm*NADP-ME).

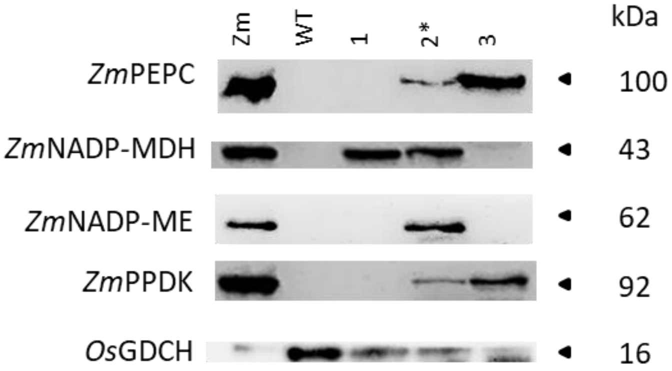

Supplementary Figure 6. Soluble leaf protein. Immunoblots for Quintuple (PEPC-28/PPDK-11/MDH-40/ME-116/*gdch*-31) crosses. Maize (Zm), wild-type rice (WT) and plants of F_1 crosses (numbers). Asterisk denotes plant in which all four proteins (PEPC, PPDK, MDH and ME) were expressed and GDCH was suppressed. Protein was extracted from the youngest fully expanded leaf at mid-tillering stage; samples were loaded on an equal leaf area (0.2364 mm^2 for *Zm*PEPC, 1.182 mm^2 for *Zm*PPDK and *Zm*NADP-MDH; and 2.364 mm^2 for *Zm*NADP-ME and *Os*GDCH).

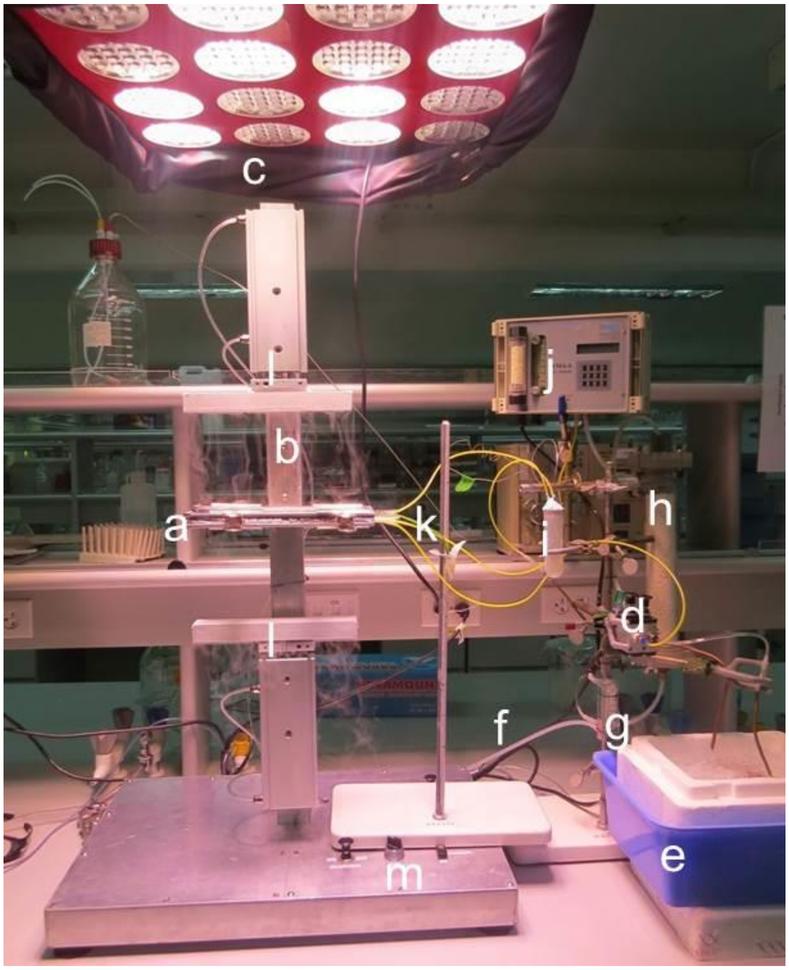

Supplementary Figure 7. Custom gas exchange freeze clamp apparatus. (a) custom gas exchange chamber, (b) mounting frame, (c) LED light bank, (d) air conditioning system control, (e) copper coil and ice bath for air cooling, (f) air inlet, (g) flow control, (h) CO_2 scrubber, (i) $^{13}CO_2$ gas delivery system, (j) CO_2 analyser (second unit not shown), (k) chamber air inlet and outlet (thermocouple not shown), (l) liquid nitrogen cooled aluminium bars, (m) pneumatic control.

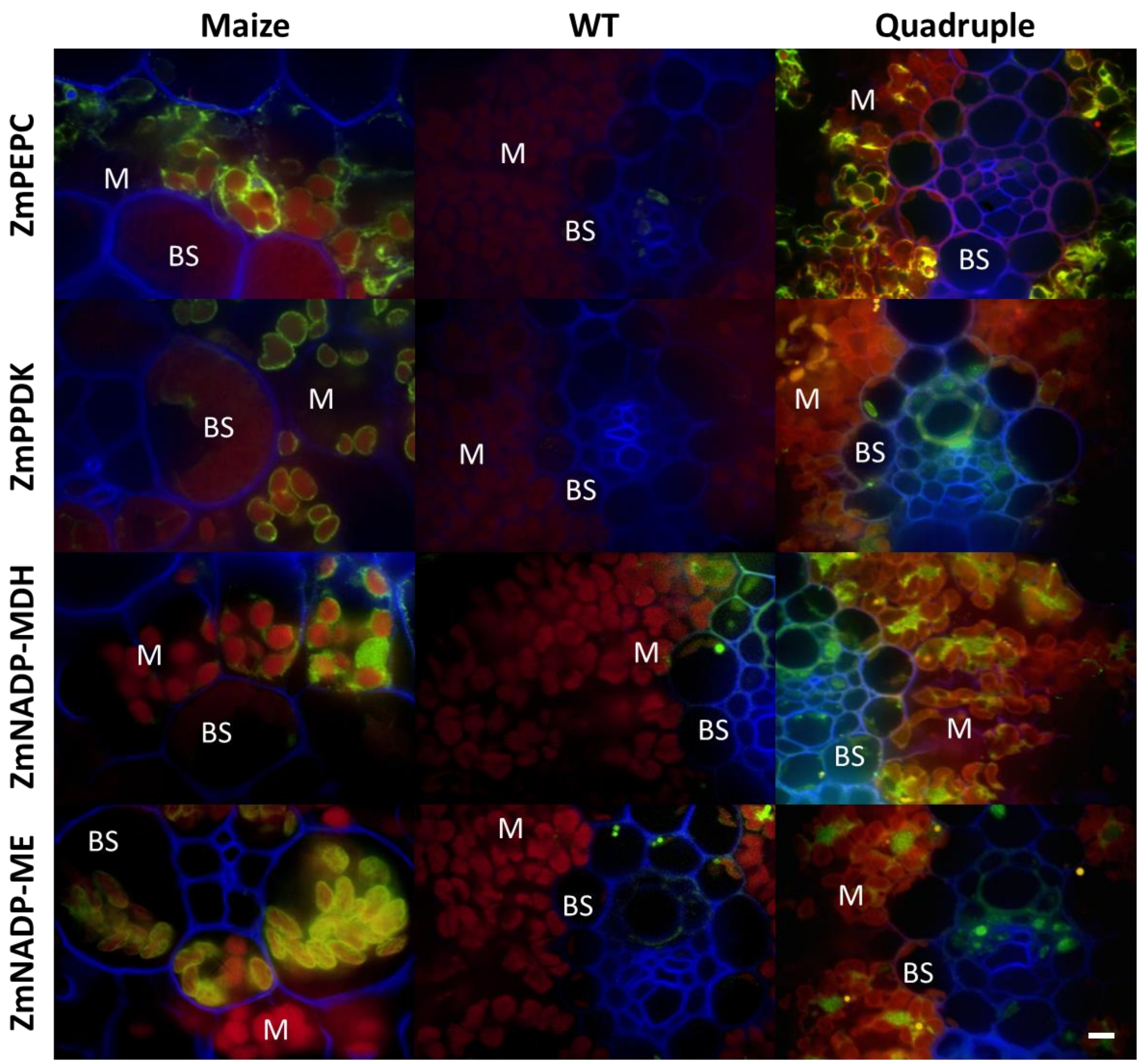

Supplementary Figure 8. Representative images of immunolocalization of *Zm*PEPC, *Zm*NADP-MDH, *Zm*NADP-ME and *Zm*PPDK protein. Anti-rabbit polyclonal antisera (*Zm*PEPC 1:500, *Zm*NADP-MDH 1:10, *Zm*NADP-ME 1:100, *Zm*PPDK 1:25) plus Alexa Fluor 488 goat anti-rabbit IgG as secondary antibody (1:200; shown in green color). Red shows autofluorescence of chlorophyll in chloroplasts. Co-staining with calcofluor white visualized cell walls (shown in blue). Scale bar: 5 µm; where M is mesophyll and BS is bundle sheath. Images are of the middle portion the seventh fully expanded fifth leaf of maize, wild-type IR64 (WT) and one representative plant for F_2 generation quadruple crosses. Maize: positive control. Wild-type: negative control.

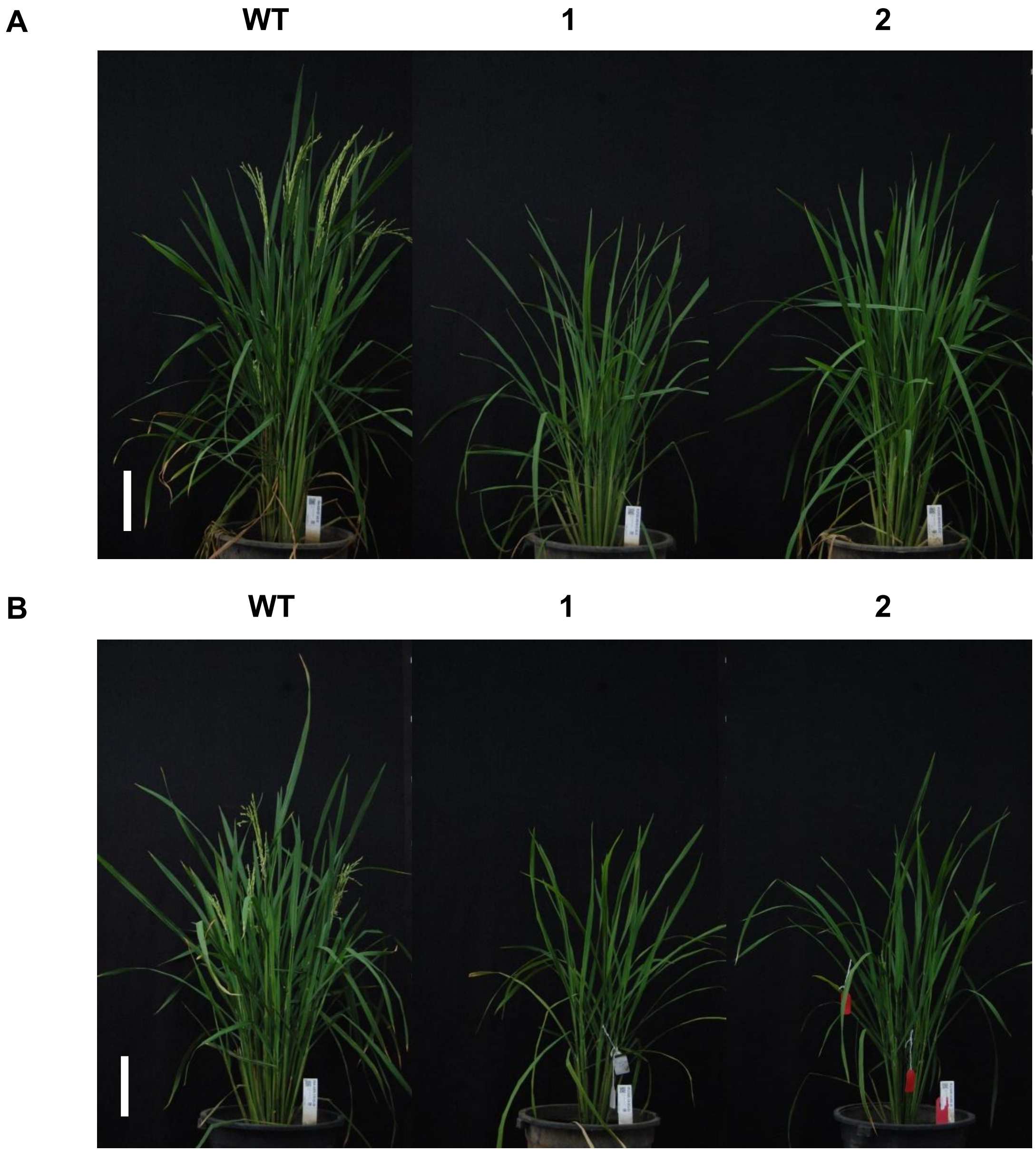

Supplementary Figure 9. Representative pictures of wild-type (WT) and two representative **(A)** quadruple and **(B)** quintuple crosses. Scale bar 10 cm. 90 days post germination (DPG).

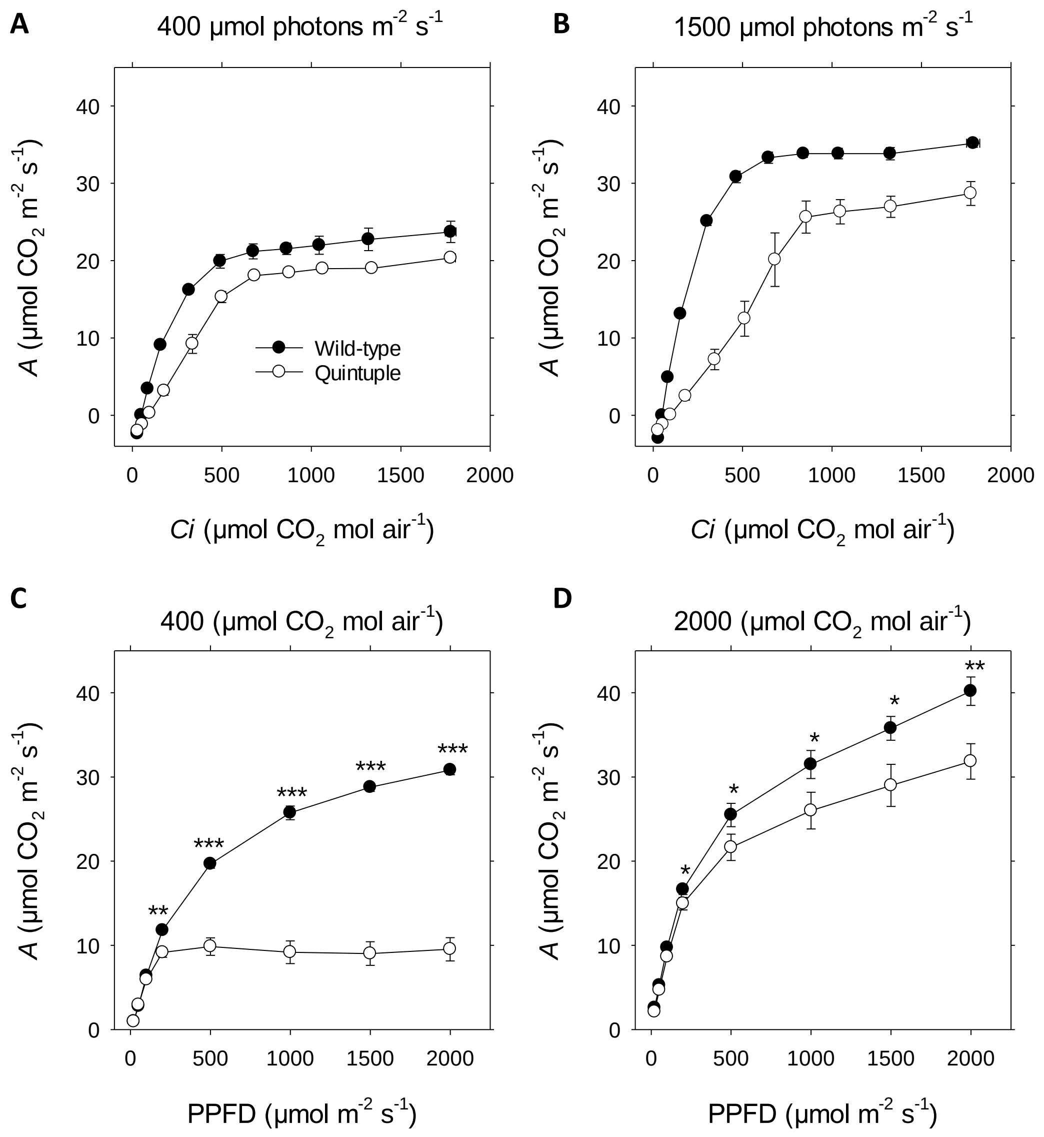

Supplementary Figure 10. (A, B) Net CO_2 assimilation rate (*A*) in response to intercellular pCO_2 (*Ci*,) and (c, d) photosynthetic photon flux density (PPFD). Measurements were made at 400 µmol photons m^{-2} s^{-1} **(A)** and 1500 µmol photons m^{-2} s^{-1} **(B)** under 21 % O_2. Measurements were made at a pCO_2 (C_a) of 400 µmol CO_2 mol air^{-1} **(C)** and 2000 µmol CO_2 mol air^{-1} **(D)**. Values are means ± SE of three individual F_2 generation quintuple crosses and four wild-type plants. A Student's t-test was performed for **(C, D)**. Significant differences between WT and quintuple within PARi level are indicated by *P-value<0.05, ** P-value<0.01, *** P-value<0.001.

Supplementary Table 1. Comparison of photosynthetic parameters.

	Γ	CE	R_d	Φ
	µmol CO_2 m^{-2} s^{-1}	µmol CO_2 m^{-2} s^{-1}µmol CO_2 mol^{-1}	µmol CO_2 m^{-2} s^{-1}	
	Low light (PARi 400 µmol m^{-2} s^{-1})		Ambient CO_2 (400 µmol CO_2 mol air^{-1})	
WT	50.26 ± 1.05^{b}	0.09 ± 0.00^{a}	0.45 ± 0.09^{ns}	0.06 ± 0.00^{a}
Quintuple	89.03 ± 11.24^{a}	0.04 ± 0.01^{b}	0.26 ± 0.12^{ns}	0.04 ± 0.00^{b}
	High light (PARi 1500 µmol m^{-2} s^{-1})		Elevated CO_2 (2000 µmol CO_2 mol air^{-1})	
WT	48.69 ± 2.16^{b}	0.13 ± 0.00^{a}	*nd*	0.08 ± 0.00^{ns}
Quintuple	95.33 ± 10.36^{a}	0.03 ± 0.00^{b}	*nd*	0.07 ± 0.00^{ns}

CO_2 compensation point (*Γ*), carboxylation efficiency (CE), respiration rates (R_d) and quantum yield for CO_2 assimilation (Φ). Measurements of Γ and CE were made at a PPFD of 400 or 1500 µmol photons m^{-2} s^{-1}. Measurement of R_d and Φ were made at a pCO_2 (C_a) of 400 or 2000 µmol CO_2 mol air^{-1} and a leaf temperature of 25°C. Values are means ± SE of three F_2 generation cross plants and four wild-type (WT) plants. Different letters indicate those values that are statistically different between WT and quintuple within pCO_2 level or PARi level based on a Student's t-test, P-value<0.05. ns indicates non-significant. *nd*, not determined.

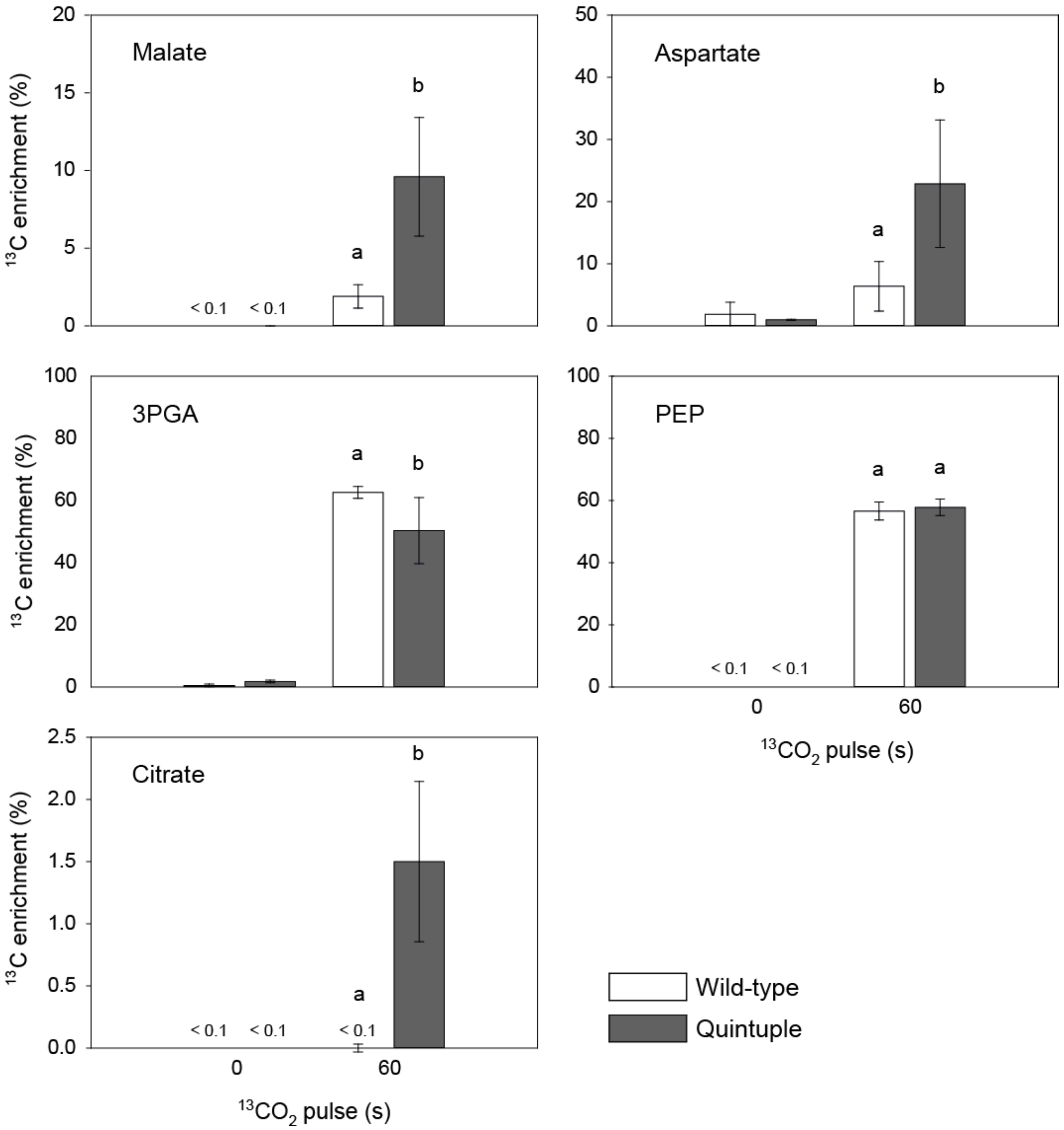

Supplementary Figure 11. $^{13}CO_2$ pulse-labelling of wild-type and quintuple rice lines. Fully expanded leaves were pulse-labelled with $^{13}CO_2$ (300 ppm) for 60 s under steady state photosynthetic conditions. Isotopomers of malate, aspartate, 3PGA, PEP and citrate were measured in extracts from pulse-labelled (60 s) and non-labelled (0 s) leaves by LC-MS/MS, and ^{13}C enrichment (%) was calculated after correction for natural abundance. Values are means ± SE of four individual F_2 generation quintuple crosses and four wild-type plants. The original data are presented in Supplementary Dataset B. Different letters within groups indicate those values that are statistically different based on a one-way ANOVA, P-value<0.05.
